# Complication rates after autologous cranioplasty following decompressive craniectomy

**DOI:** 10.1007/s00701-024-06282-w

**Published:** 2024-09-25

**Authors:** Leonard Ritter, Kilian Strohhäcker, Karl-Michael Schebesch, Thomas Eibl, Julius Höhne, Adrian Liebert

**Affiliations:** Department of Neurosurgery, Paracelsus Medical University, Breslauer Str. 201, 90471 Nuremberg, Germany

**Keywords:** Autologous cranioplasty, Decompressive craniectomy, Complications, Skull reconstruction, Cranial defect, Infection, Bone flap resorption, Risk factors

## Abstract

**Objective:**

The reimplantation of autologous bone grafts after decompressive craniectomy (DC) is still up for debate. The objective of this study was to analyze the surgical revision rate for autologous cranioplasties in our center, aiming to identify predictors for procedure-related-complications.

**Methods:**

A retrospective single-center study was conducted for adult patients who underwent autologous cranioplasty after DC. The primary endpoint was the complication rate in terms of surgical revision and removal of the bone graft: infection, new onset seizures, dislocation, haemorrhage, osteolysis, wound dehiscence and cerebrospinal fluid (CSF) fistula. Demographic data, medical records, surgical reports and imaging studies were analysed and risk factors for complications were evaluated.

**Results:**

169 consecutive patients were included. The median interval between DC and cranioplasty was 84 days. Mean age was 51 ± 12.4 years. 26 patients (15.3%) had revision surgery for following reasons. *n* = 9 implant dislocations (5.3%), *n* = 7 osteolysis (3.6%), *n* = 6 infections (3.6%), *n* = 5 had re-bleedings (3%), *n* = 5 wound dehiscences (3%), and *n* = 2 CSF fistulas (1.2%). 18 patients developed new seizures (10.7%). Bi- and multivariate analysis revealed three independent risk factors, simultaneous ventriculo-peritoneal (VP) shunting increased the risk for material dislocation (*p* < 0.001); large bone grafts (> 193.5 cm^2^) increased the risk for osteolysis (*p* = 0.001) and bifrontal cranioplasties were associated with higher risk for infections (*p* = 0.04).

**Conclusion:**

The complication rates in our study were comparable to previously reported data for autologous or artificial cranioplasties. As osteolysis was correlated to larger bone grafts, a synthetic alternative should be considered in selected cases.

## Introduction

Cranioplasty after decompressive craniectomy (DC) is a standard neurosurgical procedure that aims to restore the skull’s normal architecture and protective functions, as well as to improve cerebrospinal fluid dynamics and cerebral blood flow [17]. This is crucial for facilitating neurological rehabilitation and improving neurological outcomes [18, 24].

While there is no universally accepted gold standard, the choice of implant material typically falls to the discretion of the operating surgeon or the institution [14, 19, 23].

Despite being a routine procedure, cranioplasty carries a high complication rate, which has been linked to various risk factors [35]. Among them, the choice of material has been discussed in previous studies as a modifiable risk factor [27, 13].

Initial studies reporting on cranioplasty complication rates typically focused on a single material, finding comparable complication rates across different materials [25, 32]. However, recent meta-analyses and systematic reviews have analysed complication rates among different materials, some of those emphasizing superior outcomes of synthetic, preformed grafts with lower complication rates and beneficial properties such as increased strength and high biocompatibility [18, 2, 8, 30, 16].

In our department, autologous grafts are traditionally used and the revision rate does not appear to be increased. However, a detailed analysis of the complication rate, considering potential confounders and bias, has not been performed, yet. Consequently, it was imperative to analyse our complication rate and to compare them with the data from recent studies to reaffirm, or to ban autologous cranioplasties as standard procedure.

We sought to identify predictors of different procedure-related complications, including osteolysis, rebleeding, implant dislocation, new onset seizures and cerebrospinal fluid (CSF) fistulas.

## Methods

### Ethical standard

The study was conducted according to the Declaration of Helsinki and its later amendments. No study-specific examinations were conducted. The study design was approved by our institutional review board (IRB-2024-01).

### Inclusion and exclusion criteria

This retrospective study included adult patients who underwent primary autologous cranioplasty between 2013 and 2023. Eligible patients must have had decompressive hemicraniectomy or bifrontal craniectomy previously. In our department, autologous cranioplasty is the standard procedure after craniectomy, only after osteolysis and infection, a synthetic bone flap is used. Patients with cranioplasty following gunshot injuries were excluded from the analysis.

### Outcome assessment

The aim of our study was to identify the complication rates after autologous cranioplasty that required surgical revision and removal of the bone graft. Among them were on-site infections, new onset epileptic seizures, material dislocation, secondary haemorrhage, osteolysis, wound dehiscence and CSF fistulas. Complications were only assessed if revision surgery was performed. In some cases, more than one complication occurred in one patient. We aimed to identify individual risk factors for the respective complications.

### Data collection

Laboratory findings, demographic data, medical records, intraoperative course and imaging studies were retrospectively analysed.

The primary diagnoses for decompressive craniectomy were categorized into four groups: ischemic stroke, subarachnoid haemorrhage (SAH), traumatic brain injury (TBI) and intracranial haemorrhage (ICH). If several aetiologies were present in a patient, the patient was allocated to the dedicated group. In 31 craniectomies (18.4%), the bone flap was removed in more than one piece, so the bone fragments had to be fixed by titanium connectors before the bone craft was reinserted as one piece. The factor “dural level” describes, at which level the dura was in the relation to the bony border of the craniectomy during cranioplasty. “Above” described that the dural level protruded over the bone level. “Below” referred to intraoperative findings in which the dura was below bone level. C-reactive protein (CRP) and leucocyte counts were collected as laboratory values. The data was then dichotomized as being within the normal range (≤ 0.5 mg/dl for CRP and ≤ 10 × 10^9^/l for leucocyte count) or as being elevated. The patients’ overall status was assessed using the Charlson Comorbidity Index (CCI) and the American Society of Anaesthesiologists risk classification (ASA) [4, 7]. Operation time was measured in minutes from the initial skin incision to the completion of the suturing. The surface area of the bone craft was calculated using the AC method, in which the craniectomy length (A) is multiplied by its height (C). This method was shown to be as accurate as alternative methods like the marching cube and quasi-Monte Carlo methods in a recent study be Ho et al. [12]. A CSF fistula was defined as a visible CSF outflow from the wound that was confirmed by a Beta-2-Transferrin test. Rebleeding was defined as either epi-, subdural or intracerebral bleeding that had to be surgically revised due to a space occupying effect. Material dislocation included the displacement of the bone flap beyond the bone boundaries and/or osteosynthetic material loosening. Osteolysis was assessed using CT scans and only counted as complication if a reoperation was necessary due to progressive bone flap resorption.

### Bone flap preservation and storage

All bone grafts were stored immediately after the decompressive craniectomy using cryopreservation in deep freezer at a temperature below –80°C. Before cryopreservation, all remaining tissue were entirely removed, including pericranium, muscle, fascia, and galea. For reimplantation, the scrub nurse removed the bone graft from the sterile wrapper, washed it with warm saline, and placed it in a sterile basin container. The bone graft then remained submerged for 20–30 min at room temperature until being reimplanted.

### Operative and postoperative procedure

Part of the clinical routine of this operation is a preoperative antibiotic treatment with either cefuroxime or cefazolin 30 min before the initial skin incision.

All surgical procedures were performed by or under supervision of a certified neurosurgeon. After removing the patient’s hair with a margin from the incision line, the scalp was first cleaned of skin impurities with a swap soaked in gasoline and then cleaned with an alcoholic skin preparation. After the bone margins were fully exposed, the temporalis muscle was dissected from the dura so that the bone craft could be placed between the dura and the muscle. The cranioplasty was fixed to the bone margins either by titanium clamps or by using titanium miniplates and self-tapping screws.

Dural tenting sutures were used to adapt the dura to the bone craft to avoid epidural haemorrhage. Before closing the wound in a multilayer fashion, a subgaleal wound drain was used. At the end of the operation, the wound was closed with wound closure strips (Steri-Strips™). These remained on the wound until the 3rd postoperative day. Then a change was made to a normal plaster, which was afterwards changed as part of the daily wound check.

**Fig. 1:**
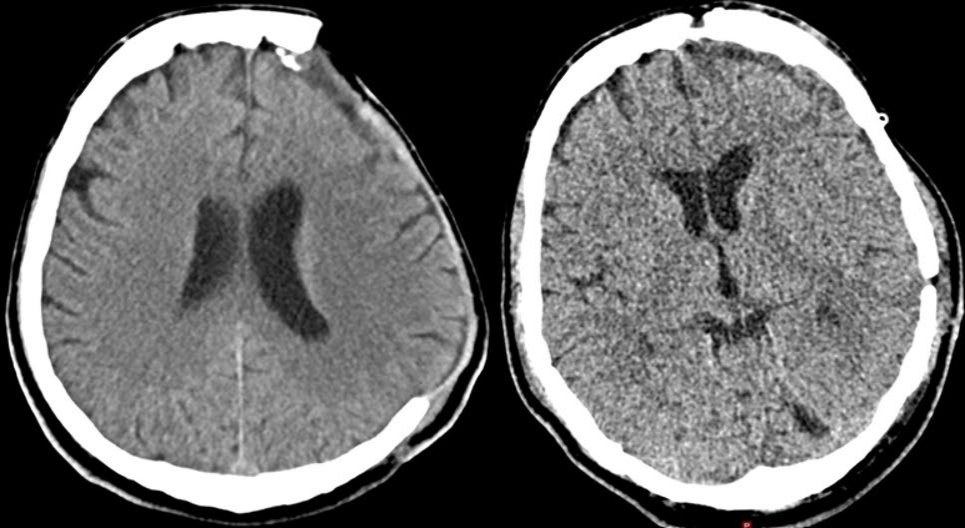
Preoperative and postoperative CT scan of a patient receiving an autologous cranioplasty

Within 24 h, a CT scan ruled out any relevant haemorrhages or brain swelling (Fig. 1). Sutures were removed on postoperative day 14. Some patients required a ventriculoperitoneal (VP) shunt either before or after the cranioplasty, depending on the severity of the hydrocephalus that had developed.

### Statistical analysis

Statistical analyses were performed using IBM SPSS® Version 29 for Windows 10 (IBM Corp. Released 2020. IBM SPSS Statistics for Windows, Version 27.0. Armonk, NY: IBM Corp). Continuous variables are presented as means with standard deviations (±SD). Categorical variables are reported as absolute numbers and percentages. Comparisons of continuous variables were conducted using the Mann-Whitney U test and Kruskal-Wallis test, while Fisher's exact test and Fisher-Freeman-Halton test were used for categorical variables. A multivariable stepwise regression analysis was done for all complications with significant correlations in the bivariate analysis. For the multivariate analysis, all factors that were significant in the bivariate analysis and the most likely factors of bias of the respective complication parameters were included. The significance level was set to p < 0.05 in two-tailed testing.

## Results

### Patient cohort

169 patients were included in this study. The mean age was 51 years (±12.4), and 72 (42.6%) patients were female. The mean CCI was 2.9 (±1.8) and the mean ASA was 2.8 (± 0.6).

The most common indication for decompressive craniectomy was ischemic stroke (34.3%), followed by TBI (31.4%), ICB (18.3%) and SAB (15.4%). Right sided craniectomy (48.5%) was more frequently done then left sided (40.2%) and bifrontal craniectomy only in 11.2% of the cases. Multiple bone fragments were used in 31 cases (18.3%).

Intraoperative CSF drainage had to be conducted in 69 (40.8%) cases and unintended dural injury occurred in 77 cases (45.6%) during the procedure. The mean operation time was 129 ± 43.7 min (median 117 min), and the mean size of the bone graft was 164 ± 55.1 cm^2^ (median 165 cm^2^). The mean time interval between craniectomy and cranioplasty was 87.2 ± 41.4 days (median 84 days).

Baseline data can be found in Table 1 and 2.

**Table 1:** Factors - nominal

Category	Factor	Value label	(*n*)	(%)
Demography	Gender	Female Male	72 97	42.6 57.4
Comorbidities	DM type 2	No Yes	147 22	87 13
	Hypertension	No Yes	76 93	45 55
	Primary diagnosis	Infarction	58	34.5
		SAB	26	15.5
		TBI	53	31.5
		ICB	31	18.5
Medication	Anticoagulation	No Yes	158 11	93.5 6.5
	Antiplatelets	No Yes	139 30	82.2 17.8
Intraoperative factors	Location of cranioplasty	Left Right Bifrontal	68 82 19	40.2 48.5 11.2
	Multiple fragments	No Yes	137 31	81.5 18.5
	Dural level	Below At level Above	58 33 59	38.7 22 39.3
	Intraoperative CSF withdrawal	No Yes	99 69	58.9 41.1
	Unintended dural opening	No Yes	91 77	54.2 45.8
	Wound drain	No 1 Day 2 Days	1 84 84	0.6 49.7 49.7
Laboratory values	CRP	≤ 0,5 mg/dl > 0,5 mg/dl	88 60	59.5 40.5
	Leucocytes	≤ 10 × 10^9^/l > 10 × 10^9^/l	136 24	85 15
Others	VP shunting before cranioplasty	No Yes	151 18	89.3 10.7
	VP shunting after cranioplasty	No Yes	152 17	89.9 10.1

*n* patient number, *DM* Diabetes mellitus, *TBI* Traumatic brain injury, *SAB* Subarachnoid haemorrhage, *ICB* Intracranial haemorrhage, *VP* Ventriculoperitoneal, *CRP* C-reactive protein

**Table 2:** Factors - metric

Category	Factor	Mean	Min/Max	SD
Demography	Age	50.9	19/74	± 12.4
Comorbidities	CCI	2.9	0/7	± 1.8
	ASA	2.8	1/4	± 0.6
Intraoperative factors	Operation time (min)	129	60/309	± 43.7
	Size of bone graft (cm^2^)	164.9	30/310	± 55.1
Others	Time interval between surgeries (days)	87.2	9/329	± 41.4

*SD* Standard deviation, *CCI* Charlson Comorbidity Index, *ASA* American Society of Anaesthesiologists risk classification

### Complications

26 patients (15.3%) had to be revised. 9 patients had implant dislocation (5.3%), 7 patients developed osteolysis (3.6%), 6 patients presented with postoperative bone flap infection (3.6%), 5 patients who suffered re-bleeding (3%), 5 patients had wound dehiscence (3%), and 2 patients presented with CSF fistula (1.2%). In addition, 18 patients developed new seizures (10.7%). The median time interval between cranioplasty and osteolysis was 28.9 month (±18.2). An overview of the complication rates is given in Table 3.

**Table 3:** Complications

Complication type	(%)
Seizures	89.3 10.7
Implant dislocation	94.7 5.3
Osteolysis	95.9 4.1
Infection	96.4 3.6
Rebleeding	97 3
Wound dehiscence	97 3
CSF fistula	98.8 1.2

Wound dehiscence and CSF fistulas occurred as concomitant complication with material dislocation and therefore did not represent a separate indication for revision surgery.

The median postoperative follow-up was 23 months.

In all cases of infection, at least one pathogen could be identified and in four patients (66.7%), two or more genus of bacteria could be cultured. The most common pathogens were Staphylococci (4), followed by Cutibacterium acnes (3), Enterobacteriaceae (1) and Finegoldia magna (1).

### Bivariate analysis

A bivariate and multivariate analysis was done for all complications except for CSF fistulas as there were two few events. In the analysis, there was a significant correlation for every complication except for new onset seizures and postoperative bleeding.

First, VP shunting after cranioplasty was correlated to the risk of material dislocation (*p* < 0.001). Furthermore, the size of the bone flaps was significantly correlated with the probability of osteolysis. Thereby the mean size difference between cases with and without osteolysis was 161 ± 53.4 cm^2^ vs 235 ± 40.9 cm^2^. The Receiver Operating Characteristic (ROC) analysis showed a good result with an area under the curve of 0.88. For a sensitivity of 100%, the positive cut-off value was a size of the bone graft of > 193.5 cm^2^ measured in the AC method in our study.

Bifrontal cranioplasties were more prone to infection (*p* = 0.009) than unilateral cranioplasties. While the pooled incidence for infection for left and right sided cranioplasty was 2%, it was 18.7% after bifrontal cranioplasty, resulting in a 9.4-fold increase in infection probability. Finally, TBI and infarction as primary diagnosis were more prone to wound dehiscence than SAH and ICH (*p* = 0.02) (Table 4).

**Table 4:** Bivariate analysis of potential risk factors for complications

Category	Factor	Seizures	Implant dislocation	Osteolysis	Infection	Rebleeding	Wound dehiscence
Demography	Gender	0.4	0.3	0.64	0.71	0.07	0.43
	Age	0.12	0.61	0.09	0.56	0.99	0.25
Comorbidities	DM type 2	0.06	0.61	0.34	1.0	0.51	0.51
	Hypertension	0.58	0.19	0.8	0.69	0.38	0.82
	CCI	0.1	0.17	0.96	0.71	0.59	0.98
	ASA	0.14	0.58	0.46	0.62	0.9	0.95
	Primary diagnosis	0.93	0.51	0.46	0.24	0.5	**0.02**
Medication	Anticoagulation	0.61	0.42	0.51	0.34	0.55	0.29
	Antiplatelets	0.01	0.66	0.94	0.59	0.89	0.59
Intraoperative factors	Location of cranioplasty	0.74	0.96	0.74	**0.009**	0.56	0.11
	Multiple fragments	0.84	0.67	0.91	0.14	0.23	0.93
	Dural level	0.97	0.67	0.62	0.26	0.32	0.05
	Intraoperative CSF withdrawal	0.48	0.49	0.19	0.40	0.33	0.65
	Dural injury	0.53	0.93	0.84	0.1	0.18	0.38
	Wound drain	0.05	0.1	0.98	0.43	0.67	0.64
	Operation time (min)	0.62	0.91	0.08	0.46	0.55	0.14
	Size of bone graft (cm^2^)	0.66	0.66	**0.002**	0.37	0.05	0.85
Laboratory values	CRP	0.69	0.86	0.98	0.08	0.65	0.08
	Leucocytes	0.008	0.84	0.29	1.0	0.34	0.56
Others	VP shunting before cranioplasty	0.95	0.96	0.63	0.5	0.43	0.44
	VP shunting after cranioplasty	0.13	** < 0.001**	0.58	1.0	0.45	0.45
	Time interval between surgeries (days)	0.38	0.05	0.11	0.61	0.47	0.83

*DM* Diabetes mellitus, *SAH* Subarachnoid haemorrhage, *ICH* Intracerebral bleeding, *VP* Ventriculoperitoneal, *CRP* C-reactive protein

### Multivariate analysis

After the bivariate analysis, a multivariate analysis was performed for all complications except CSF fistula new onset seizures and rebleeding as there was no significant correlation found to any factor in the bivariate analysis. The multiple regressions confirmed most of the results of the bivariate analysis except for wound dehiscence, where there was no longer a significant correlation with the primary diagnosis in the multivariate analysis (Table 5).

**Table 5:** Independent risk factors for specific complications

Factor	Complication	β	SE	*P*-value	95% CI
VP shunting after cranioplasty	Implant dislocation	0.36	0.07	** < 0.001**	0.14 – 0.4
Seize of bone graft	Osteolysis	0.001	0.05	**0.001**	0.23 – 0.02
Location of cranioplasty	Infection	0.18	0.03	**0.04**	0.01 – 0.1

*P*-value was calculated by using multiple linear stepwise regression with the respective significant results of the bivariate analysis as well as the possible confounders of the individual complications. All regressions included demography, comorbidities, primary diagnosis and mediation

*β*, standardized coefficient beta, *SE* standard error, *CI* Confidence interval

## Discussion

### Complication rates

New seizures were the most common complication, occurring in over 10% of patients after autologous cranioplasty. Comparing this result to existing literature is challenging due to significant variations in reported postoperative seizure rates. Yeap et al. and Hirschmann et al. reported high incidences of 26.5% and 23.2%, respectively [11, 34]. In contrast, a systematic review by Yao et al. found a pooled incidence of only 0.043% [33]. Spencer et al. reported a pooled incidence of 5.1%, which is closer to our findings [29]. These discrepancies likely arise from study heterogeneity and bias due to various risk factors. Overall, our results fall within the reported range for epileptic events after cranioplasty.

Bone graft loosening had a prevalence of 5.3% in our study, which seems high compared to other studies reporting rates of 1% or less [30, 10]. This discrepancy may arise because implant dislocation often occurs with other complications (e.g., rebleeding, wound dehiscence, CSF fistulas) and is then classified differently or not recorded. Consequently, comparing our data with previous studies is challenging.

The rate of osteolysis was 4.1%, with a median interval of about 29 months from implantation to explanation. Several studies report higher bone flap resorption rates after autologous cranioplasty, exceeding 10% [19, 30]. However, some studies, like Schuss et al., show a similar rate of around 4% [26]. The variation could be due to different follow-up lengths, as Park et al. established a positive correlation between osteolysis rate and the length of follow-up [22].

The postsurgical infection rate was 3.6%, with Staphylococci species being the most common pathogens. This rate is slightly below the 5–10% range described in the literature [19, 1, 5, 15]. The overall infection rate does not seem related to the cranioplasty material used [19, 32, 3]. Comparability is limited by varying infection definitions, as many authors only counted infections requiring revision surgery. Additionally, not all studies administered prophylactic antibiotics, which may have skewed the average infection rate [30].

Secondary bleeding requiring reoperation for hematoma evacuation occurred in 3% of cases. Pfnür et al. reported a pooled postoperative hematoma rate of 7.9% in their retrospective analysis [23]. Klinger et al. found a 3.5% revision rate for epidural hematomas, similar to our incidence [15]. Zanaty et al. reported a 6.9% surgical site hematoma rate, with all patients needing reoperation [35]. Malcolm et al.'s systematic review found a 5.9% rate of hemorrhagic complications without specifying reoperation rates [18]. Overall, our reoperation rate due to rebleeding is within the range of other reported results.

In addition to the more common complications, we observed wound dehiscence in 3% of cases and CSF fistulas in 1.2%. Comparing these results with recent large systematic reviews, we found similar prevalence rates for these rarer complications.

Comparability with our results to previous studies was overall limited due to varying definitions and the lack of standardized reporting for surgical procedures, outcomes, and graft materials, leading to a wide range of reported complication rates in the literature.

When taking these limitations into account, our complication rates are comparable to previously published data for both autologous and other material cranioplasties. Therefore, based on the complications rates in our study, a general continued use of autologous cranioplasties is justified.

### Risk factors for complications in bivariate and multivariate analysis

Using bivariate and multivariate analyses, we aimed to identify risk factors for certain complications and determine specific indications where autologous cranioplasties might be problematic. We identified three risk factors for different complications: VP shunting after cranioplasty increased the risk of bone graft loosening, large bone grafts increased the risk of osteolysis, and bifrontal cranioplasty was associated with a higher risk of infections.

One possible explanation for material loosening is the frequent movement of the patient's head during VP shunt placement, causing mechanical stress on the cranioplasty. A potential solution is to perform VP shunting only after the bone graft has properly integrated. If this is not feasible, securing the bone graft with additional plates could help to withstand higher mechanical loads.

Previously published data support an increased infection rate for bifrontal bone grafts, showing a correlation between bifrontal cranioplasty and higher infection rates regardless of the graft material [5, 9, 31]. De Bonis et al. found not only a higher infection rate but also an overall increase in complications after bifrontal cranioplasty, speculating that this may be due to the proximity of the frontal sinus, which could be a contamination source during and after surgery [5]. Since infection rates are higher in bifrontal cranioplasties regardless of the material used, synthetic materials likely won't reduce the infection risk. A possible optimization could be achieved by placing the subsequent cranioplasty in the clean-contaminated risk category when the frontal sinus is opened during the initial operation and adding an additional antibiotic prophylactic cover as in transnasal procedures [20]. A second possible option for reducing the risk of postoperative wound infection would be a preoperative personalized antibiotic treatment based on the results of nasal or rectal swabs. By doing so, Peredes et al. were able to achieve a significant reduction in postoperative complication rates in a study they did in 2020 [21].

Several studies have identified predictors of osteolysis in autologous cranioplasties [19, 6, 28]. However, our study is the first to describe a correlation between bone graft size and the risk of osteolysis. Since the size of the bone flaps is determined by the initial operation, this factor cannot be modified. Previous studies have shown that synthetic materials have a significantly lower risk of disintegration, suggesting that synthetic alternatives might be more suitable for large bone flaps [19, 2, 30]. Further studies are needed to confirm our findings. Unlike Di Rienzo et al., we did not find multiple bone fragments to be correlated with an increased risk of osteolysis [6].

## Strengths and limitations of the study

Our present study is inherently limited by its retrospective observational design. Another limitation is the lack of a control group with another graft material.

The strength of this study arises through the fact, that in addition to recording complications of autologous cranioplasties, several specific risk factors that are associated with the complications were identified. Additionally, concrete suggestions on how to achieve a reduction in the incidence of these complications were made. Overall, this results in a manuscript of great practical value for daily clinical practice.

## Conclusion

The overall complication rates in our study are comparable to recent data for both autologous and synthetic cranioplasties, supporting the continued use of autologous cranioplasties. However, we identified three independent risk factors: VP shunting after cranioplasty increased the risk of bone graft loosening, large bone grafts increased the risk of osteolysis, and bifrontal cranioplasty was associated with a higher risk of infections. Since the risk of osteolysis in large bone grafts cannot be modified, synthetic alternatives might be preferable in these specific cases. Further studies with larger patient numbers are needed to verify our results and identify additional risk factors.

## Data Availability

The datasets used and analysed during the current study are available from the corresponding author on reasonable request.
